# Spectroscopic Investigations of the Surface-Level Interaction of Europium (III) Ions and Polyaniline Particles

**DOI:** 10.1007/s10895-026-04897-z

**Published:** 2026-07-30

**Authors:** C. E. Secu, E. Matei, M. Baibarac, C. Negrila, M. Secu

**Affiliations:** National Institute for Materials Physics (NIMP), Bucharest-Magurele, Romania

**Keywords:** Polyaniline particles, Eu^3+^ organo-metallic complex, Eu^3+^ luminescence

## Abstract

We investigated the surface-level interaction of the Eu^3+^ ions with polyaniline (PANI-EB) particles prepared by using liquid-phase impregnation method with formation of PANI/EuCl_3_ complex hybrid structure. Scanning electron microscopy (SEM) confirmed that the micro-sized PANI-EB particles possess a porous, granular structure. X-ray photoelectron spectroscopy (XPS) revealed the Eu^3+^ ions interaction with the imine and amine groups of the PANI-EB. The Raman and FTIR spectra analysis are consistent with interaction between PANI-EB and EuCl_3_ with formation of the Eu-N bonds. Under UV excitation, the base PANI-EB polymer emission at 420 nm is accompanied by the characteristic red luminescence peaks of Eu^3+^ ions, albeit with a relatively low efficiency of approximately 4%. These results indicate the formation of a PANI/EuCl_3_ complex hybrid structure at the particle surface; its luminescent properties are attributed to a surface-mediated energy transfer within the complex via the “*antenna effect*”.

**Supplementary Information:**

The online version contains supplementary material available at https://doi.org/10.1007/s10895-026-04897-z.

## Introduction

Polymers doped with rare-earth (RE) luminescent ions have drawn significant attention as novel materials for photonic applications [1, 2], due to their functional versatility and ease of processing into various forms, such as bulk materials, films, sheets, rods, and fibers. Consequently, the design of new lanthanide coordination compounds has become a focal point for enhancing the luminescent properties of these ions. Because rare-earth salts cannot be directly doped into an organic matrix, they are typically first encapsulated by organic ligands before being dispersed into the polymer [3], where the resulting complexes are often linked to the polymer matrix via covalent bonds [4]. The luminescent performance of these systems is governed by the *‘antenna effect*,’ a process in which photo-excited energy is transferred from the covalently bonded ligands to the rare-earth ions [5].

Polyaniline (PANI) is one of the most studied conducting polymers because of the electronic, electrochemical and optical properties suitable for various applications such as sensors, optoelectronic devices, and photonic devices [6]. Recent studies on RE-ions doped PANI has revealed novel structural and optical properties or improved ones. It was shown that Eu^3+^ ions may act as structure-directed agent inducing the formation of network-like nanofiber structure [7]. The investigation of the interaction of PANI with RE has indicated the formation of a conductive complex of EuCl_3_–PANI and the proposed doping mechanism involves oxidation of PANI-EB and pseudo-protonation steps [8]. Further studies on RE-doped (PANI) with RE= Eu^3+^, Tb^3+^, Y^3+^ ions have shown an increase of conductivity because of the changes induced in the electronic structure of PANI [9]. The doping induces a highly ordered PANI structure most likely due to the RE ions cross-linking with adjacent PANI chains. The interaction between PANI and RE ions was revealed by the splitting of the PANI-EB characteristic broad luminescence band at 415 nm into a 405 and 418 nm doublet [10]. In addition, the formation of complex structures is responsible for luminescence properties assigned to an energy transfer from the organic moiety of PANI to RE ions by antenna effect [10] but the energy transfer efficiency is relatively low.

To date, investigations have primarily focused on the novel properties resulting from the rare-earth (RE) doping of bulk PANI, where ions are incorporated during the initial synthesis stage [8–10]. Conversely, while surface-level interactions—such as covalent bonding or ionic attraction to functional groups—are known to occur, research into these specific interfacial phenomena remains limited.

Motivated by the optical functionalization of polymeric materials and the surface-level interaction of the Eu^3+^ ions with polyaniline emeraldine base (PANI-EB) particles, this investigation provides further insights through a detailed, multi-technique spectroscopic analysis. This approach includes Raman scattering, X-ray photoelectron spectroscopy (XPS), and electron paramagnetic resonance (EPR), with a particular focus on luminescence properties.

## Experimental

### Samples Preparation

Aniline (C_6_H_5_NH_2_, ANI), potassium dichromate (K_2_Cr_2_O_7_), hydrochloric acid (HCl), ammonium hydroxide (NH_4_OH), acetonitrile (CH_3_CN), europium (III) chloride hexahydrate (EuCl_3_-6H_2_O), and dimethylformamide (HCON(CH_3_)_2_, DMF) were purchased form Sigma-Aldrich company.

For the PANI-EB preparation we used a mixture consisting of ANI [(1.022 g, 1.09 × 10^− 2^ mol) in 25 ml of 0.5 M HCl] and K_2_Cr_2_O_7_ [(0.56 g, 1.9 × 10^− 3^ mol) in 25 ml of 0.5 M HCl] solutions left standing under ultrasonication for 2 h at 0 °C. The formation of PANI-ES form is revealed by the green color of the suspension, which was further filtered and the resulting filter cake was washed with 1000 ml of deionized water. The transformation of the PANI-ES salt form into its base form, i.e., (PANI-EB) was reached during interaction with 500 ml 1 M NH_4_OH, followed by filtration. The oligomers were extracted with 400 ml of CH_3_CN and the remaining powder was dried at 35 °C under vacuum [11]. For the Eu^3+^-doping of the PANI-EB we used the impregnation process where a EuCl_3_-6H_2_O crystalline powder was mechanically mixed with PANI-EB polymer (ca. 25 wt%) with a ball mill. The resulting mixture was used to prepare a PANI- EuCl_3_-6H_2_O in DMF solution (ca.25wt.%) followed by the stirring for 2 h. At the end, the undissolved Eu^3+^-doped PANI-EB particles were filtered by using a filter paper (cellulose, particle retention 4–10 μm) and collected (on the filter paper) for further measurements; undoped PANI-EB particles were prepared by using the same procedure.

### Experimental Methods

The morphology of the samples was studied by using a Zeiss Gemini 500 scanning electron microscope (SEM) and equuiped with energy dispersive X-ray system (EDX) for the chemical composition analysis. The X-ray photoelectron spectroscopy (XPS) analysis, was performed by using a multi-analysis SPECS system dedicated to surface analysis, with monochromatic Al- target X-ray tube source (Al K_α_ −1486.7 eV), PHOIBOS 150 analyzer and flood gun Specs FG15/40 for charge compensation. The XPS spectra were recorded using a Pass Energy of 10 eV, whereas the extended spectrum was recorded using a Pass Energy of 50 eV. Spectral analysis and peak fitting were performed using Spectral Data Processor (v. 2.3) software, employing Voigt functions and spectrometer-specific sensitivity factors for quantitative evaluation. The energy scale was calibrated by referencing the C 1s peak—associated with C–C and C–H bonds—to a binding energy of 284.8 eV. For doublet lines resulting from spin-orbit splitting, the area ratios were constrained to their theoretical values, while the binding energy separations were obtained from the NIST XPS database [12].

For the Raman spectroscopy investigations, we used a FT Raman spectrophotometer, MultiRam model, from Bruker, operating at wavelength excitation of 1064 nm. The FTIR spectra were recorded by using a Fourier Transform Infra-Red (FTIR) spectrometer PerkinElmer BX Spectrum II spectrometer (in ATR configuration), in the 450 to 4500 cm^− 1^ range with a resolution of 4 cm^− 1^ and using 32 scans/experiment.

The photoluminescence spectra (PL) and PL lifetimes decays were recorded at room temperature by using a commercial FluoroMax 4P spectrometer; the spectra were recorded with 1 nm step and 0.5 s integration time. The Electron Paramagnetic Resonance (EPR) spectroscopy measurements were carried out at room temperature in the X-band (9.86 GHz frequency) by using a continuous-wave (CW) EMXplus EPR spectrometer equipped with a standard X-band resonator.

## Results and Discussion

**Fig. 1:**
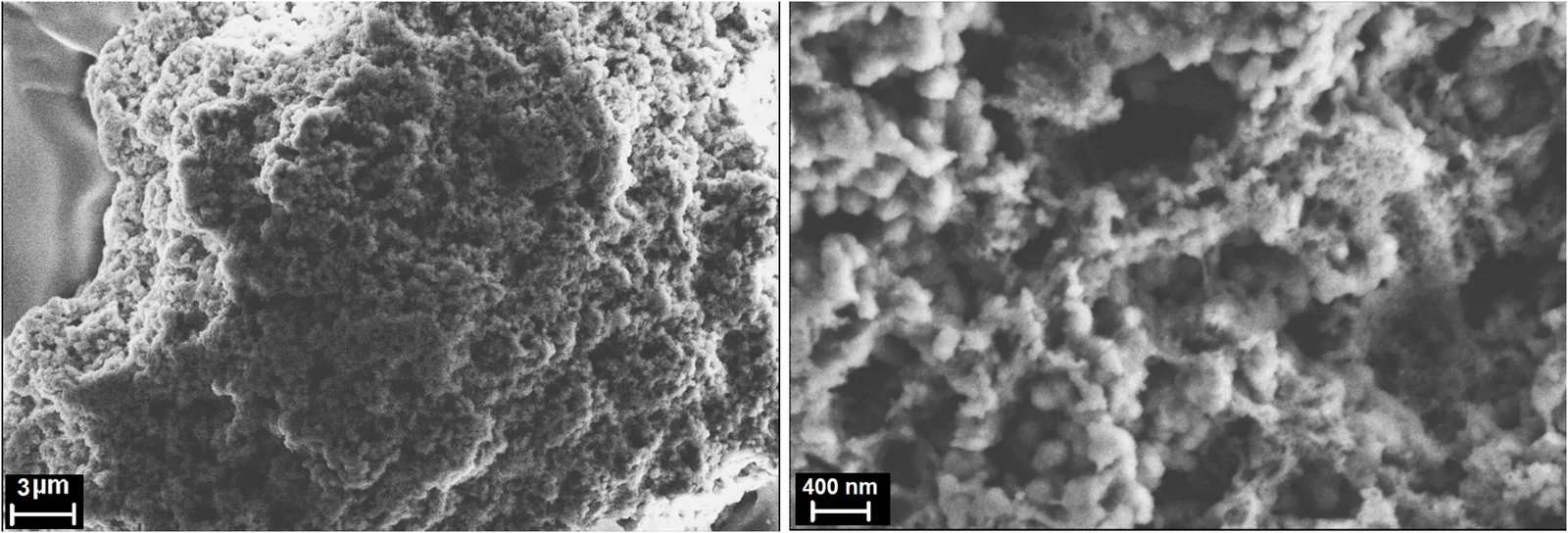
Electron microscopy (SEM) images of “as-prepared” PANI-EB particles recorded at different magnifications

**Fig. 2:**
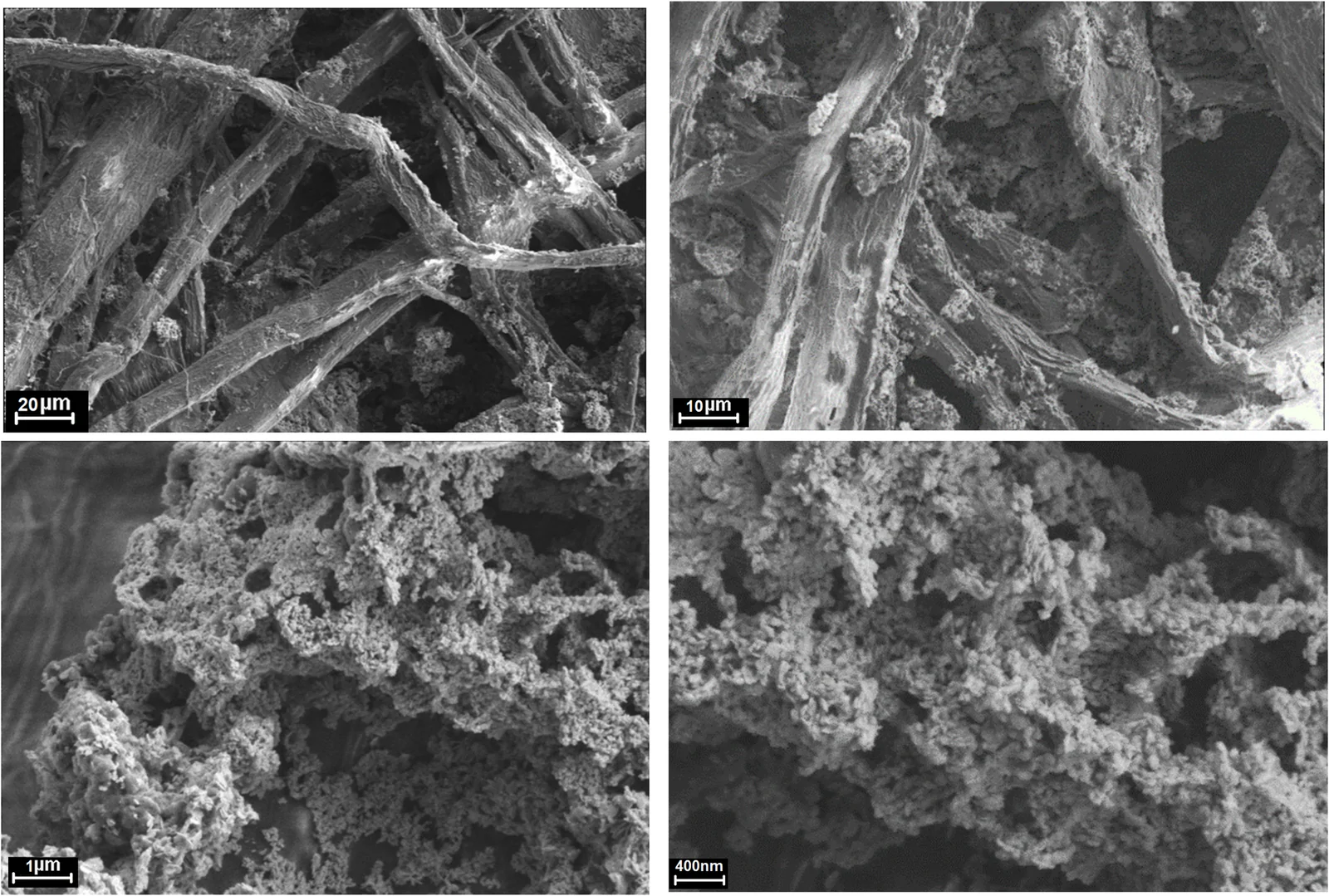
Electron microscopy (SEM) images of Eu-doped PANI-EB particles attached on the cellulose fibers recorded at different magnifications

The scanning electron microscopy (SEM) images of PANI-EB particles “as-prepared” and attached on the cellulose fibers are presented in the Figs. 1 and 2; porous, granular structure of the particles is observed. The EDX spectral analysis showed the presence, uniformly dispersed of the elements from the precursor reagents: 64 C-11O-7.0 N-12Cl-6Eu (at%) - see Supplementary information (Fig. 1S).

**Fig. 3:**
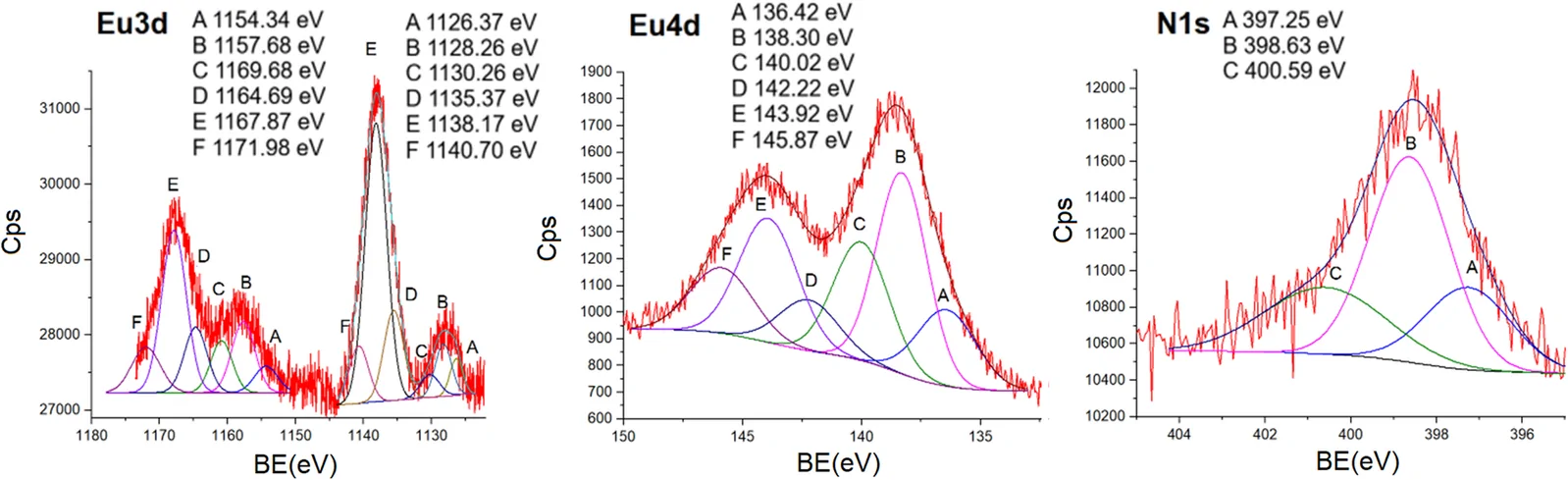
The XPS spectrum shown in the region of the Eu3d, Eu4d and N1s lines energy peaks

The **XPS** technique is recognized as a valuable investigation tool for the surface composition (of around 10 nm depth) ([13] and references therein). In order to confirm the presence of Eu^3+^ ions and to shed light on the chemical bonds and environments we carried out detailed XPS scans in the regions corresponding of the Eu3d, Eu4d lines and N1s line (Fig. 3).

The XPS spectrum in the region between 1120 and 1180 eV comprises several convoluted peaks of the **Eu3d line**: Eu3d^5/2^ and Eu3d^3/2^ at lower and higher energies, respectively. The fitting show three doublets, as it follows. The 1135.57 and 1164.69 eV peaks doublet (D, D) was assigned to the oxygen related bonds: Eu-O (as in Eu_2_O_3_) and also O-Eu-N bonds [14, 15]. The 1140.70, and 1171.98 eV doublet (F, F) is due to the Eu-Cl bonds (as in EuCl_3_) and the peaks doublet (E, E) at 1138.17, and 1167.87 eV is most probably related to O-Eu-Cl bonds (as in oxychlorides). Other shake-down satellites of the doublets are observed at 1126.37, 1154.34 eV, 1128.26, 1157.68 eV and 1130.26, 1160.88 eV. The energy region between 132 and 150 eV was assigned to the convolution of the Eu4d^5/2^ and Eu4d^3/2^ peaks of the **Eu4d line** separated by 5.7-5.8 eV and were fitted with three doublets. The 136.42, 142.22 eV doublet (A, D) was assigned to the oxygen related bonds: Eu-O (as in Eu_2_O_3_) and most probably O-Eu-N (as in oxynitrides) [14, 15]. The 140.02, and 145.87 eV doublet (C, F) is due to the Eu-Cl bonds (as in EuCl_3_) and the 138.30, 143.92 eV doublet (B, E) is likely related to O-Eu-Cl bonds. The **N1s line** is lying in the energy region between 393 and 405 eV and is composed by a low energy 397.25 eV peak assigned to the Eu-N bonds accompanied by two other ones at 398.63 and 400.59 eV assigned to the organic species with N-C and N-H bonds [15]. Consequently, XPS provides evidence of Eu^3+^ ion interactions with the imine and amine groups of the PANI-EB chains possible via coordinative bonds (with partial ionic and covalent character)-see below. Furthermore, the presence of other various europium-related bonds (e.g., Eu-O, Eu-Cl, and O-Eu-Cl)) in different chemical environments suggests the occurrence of complex structures (knowing the preference for higher coordination numbers, i.e. 8–10).

Regarding the occurrence of the Eu^2+^ ion species we looked closely on the Eu4d XPS spectrum which is well correlated with the Eu3d XPS spectrum. As the Inelastic Mean Free Path (IMFP) of the electrons of the Eu4d electrons is higher than of the Eu3d electrons, the Eu^2+^ species as supposed to be observed much better the Eu4d spectrum, since the interaction with the atmosphere is weaker for deeper layers of the samples. However, in the Eu4d spectrum (in the range below 132 eV) we have not observed the characteristic peaks of Eu^2+^ ions peaks [16] the energy values being observed at much higher values, specific only to Eu^3+^ ions.

**Fig. 4:**
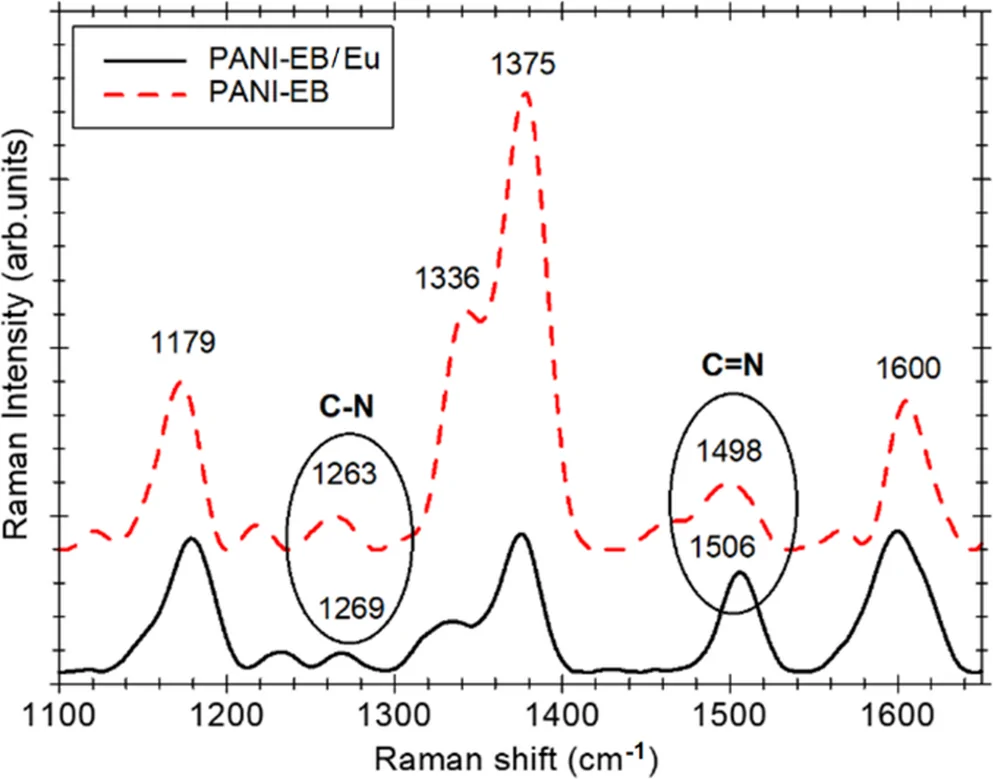
Raman spectra of PANI-EB (dotted red curve) and Eu^3+^-doped PANI-EB particles (black line curve)

The EPR spectra of the dried Eu3+-doped PANI-EB particles exhibit a single, well-defined line at 3520 G with a g-value of 2.0015 (Fig. 2S). As reported in [17], this signal is assigned to delocalized charge carriers with spin (s = 1/2) in the PANI matrix, known as polarons. The ubiquitous U-type spectrum associated with presence of Eu^2+^ ions [18] was not observed. This indicates that the coordination of Eu^3+^ ions to the imine nitrogens of the PANI-EB matrix is not accompanied by a redox reaction between the metal ions and the polymer to form Eu^2+^ species [9]. Further evidence of the interaction between PANI-EB and Eu^3+^ via covalent-coordinative bonds is provided by the Raman spectroscopy investigations presented in Fig. 4. The main Raman lines of Eu^3+^-doped PANI-EB located at ca.1179, 1269, 1375, 1486 and 1600 cm^− 1^ are assigned to the vibration modes of the polymeric chain: C–H bending (Q)-Ag mode, C–N stretch.+ring def. (B) + C–H bending (B), C–C stretch. (Q) + C–H bending(B), C = N stretch.+C–H bending (B) and C–C stretch.(B) + C = C stretch.(Q), respectively [19]. The spectra of PANI-EB and Eu^3+^-doped PANI-EB shows no major difference indicating there is no distortion of the polymeric structure. However, a close look in the 1520–1650 cm^− 1^ region expected for the vibration modes of the C-N (imine) si C = C has revealed an up-shift of the Raman lines assigned to the C–N stretch.+ring def. (B) + C–H bending (B) vibration mode from 1263 to 1269 cm^− 1^ and for the C = N stretch.+C–H bending (B) vibration mode from 1500 to 1506 cm^− 1^; the energy shift are quite small of about few cm^− 1^ but still are lying in the range observed for the C-N and C = N bonds.

**Fig. 5:**
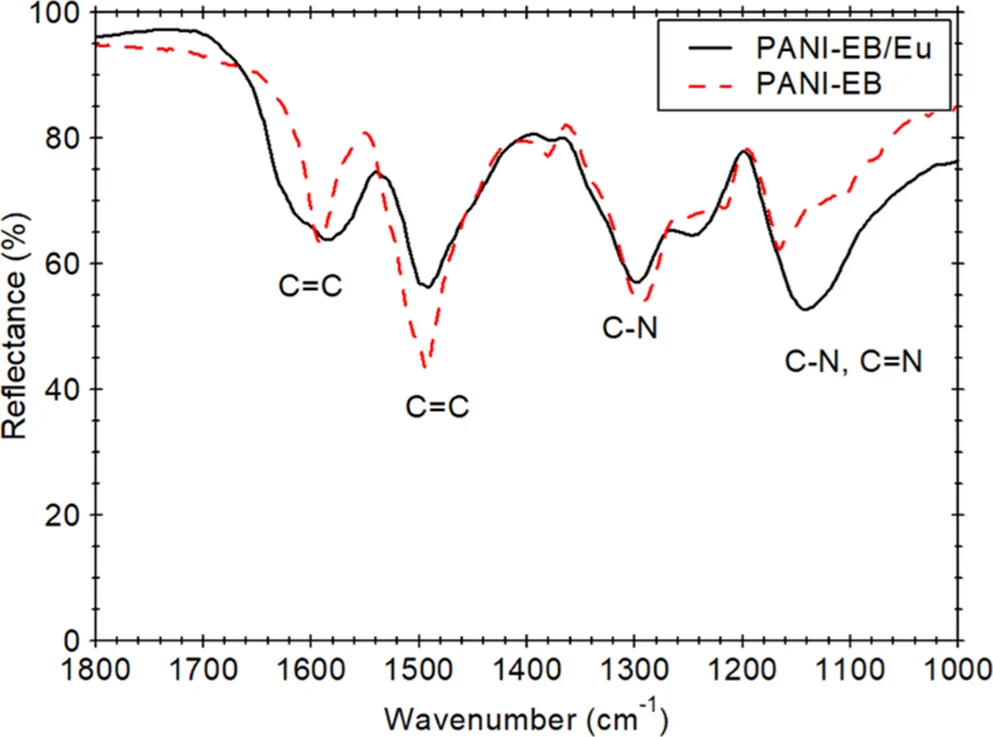
FTIR spectra of PANI-EB (dotted red curve) and Eu^3+^-doped PANI-EB particles (black line curve)

Additional evidences for the Eu-N bonds occurrence are provided by the FTIR measurements presented in the Fig. 5. The spectra primarily feature alternating bonds of the benzenoid (amine) and quinoid (imine) rings; the characteristic bands of the C-N, C = N, C = C are observed in the 1000–1800 cm⁻¹ range. The C = C stretching of the quinoid ring at 1593 cm⁻¹ peak is accompanied by to other peaks at 1493 and 1296 cm⁻¹ which are associated to the C = C stretching of the benzenoid ring and C-N stretching of the aromatic amine, respectively. The peaks observed in the 1100–1150 cm⁻¹ region are assigned to the stretching mode of the -C-N and -C = N groups in the polymer backbone, linking the benzenoid and quinoid rings. The quinoid ring stretch at 1593 cm^− 1^ is coupled to the neighboring conjugated C = N imine bonds ((N = Q=N)) and the lone pairs of electrons on the imine nitrogen (-N=) interact with the Eu³⁺ metal centers and alters the electron density redistribution along the C = N and C = C bonds. Therefore, the broadening effect observed for Eu-doped sample is the consequence of doping through the formation of Eu-N bonds. Similar broadening was observed for the -C-N and -C = N groups of the benzenoid rings (~ 1100–1150 cm⁻¹) but not for benzenoid rings (~ 1300 cm⁻¹) meaning that Eu^3+^ ions do not alter drastically the structural properties of polymer backbone.

Hence, the spectroscopic data are consistent with interaction between PANI-EB and EuCl_3_ with formation of the Eu-N bonds, most likely Eu–NR₂ covalent-coordinative bonds as was observed for polyaniline/PbI_2_ composite [11]; additional contributions such as electrostatic interactions, protonation effects, or chloride-influenced surface chemistry or local structural rearrangements of PANI-EB caused by doping cannot be excluded.

**Fig. 6:**
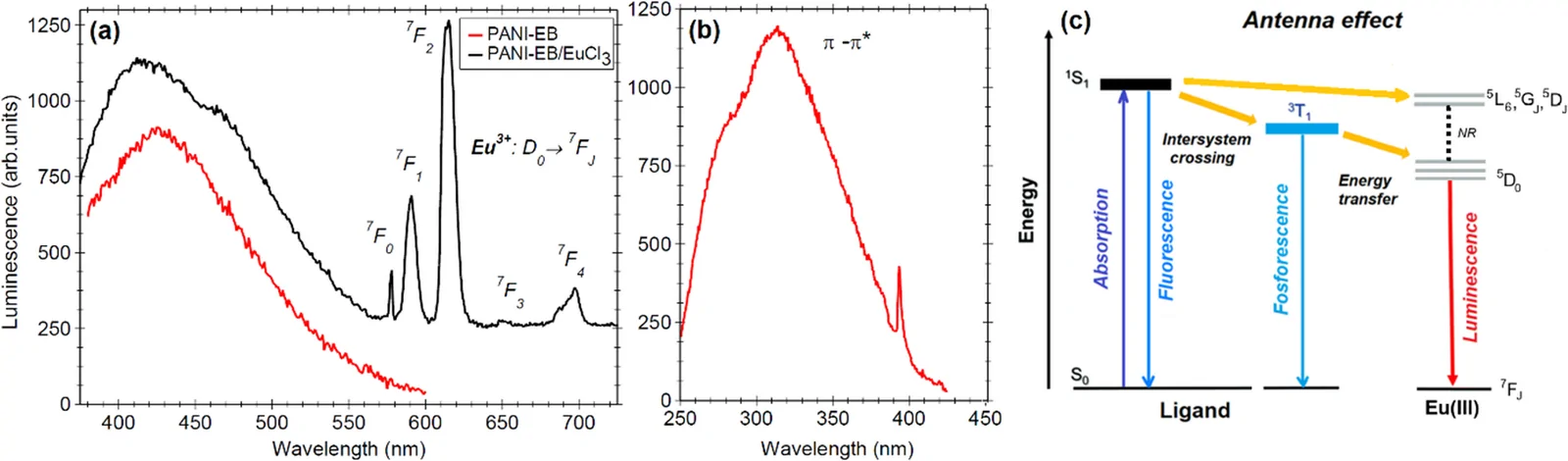
Photoluminescence spectra recorded under 325 nm excitation of undoped and Eu^3+^-doped PANI-EB (**a**) and the excitation spectrum monitoring the Eu^3+^ luminescence at 615 nm (**b**); a scheme of the “*antenna effect*” is shown (**c**)

Photoluminescence spectroscopy investigations of the Eu^3+^-doped PANI-EB presented in the Fig. 6 supported the PANI-EB interaction with EuCl_3_. Under 325 nm excitation PL spectra recorded on PANI-EB are showing broad blue luminescence peaking at about 425 nm. The 325 nm excitation peak corresponds to electronic transition π -π* of the benzenoid rings, observed for PANI-EB [20] and therefore the luminescence was assigned to the aromatic group. By contrast, the PL spectra of PANI-EB exhibit a blue broad doublet peaking at 415 and 465 nm and this spectral feature was associated with the distortion of the PANI-EB structure [11], consistent with a direct interaction with the Eu^3+^ ions.

In addition, new features are observed in the visible range where the PL spectra are showing the typical Eu^3+^ luminescence peaks at 578 nm (^5^D_0_→^7^F_0_), 591 nm (^5^D_0_→^7^F _1_), 614 nm (^5^D_0_→^7^F_2_), 650 nm (^5^D_0_→^7^F_3_) and 697 nm (^5^D_0_→^7^F_4_) transitions (Fig. 6-a). As the 325 nm excitation wavelength is outside the absorption of Eu^3+^ ions their luminescence was assigned to the sensitization by the polymer. Moreover, the excitation spectrum of the Eu^3+^ luminescence from 614 nm (^5^D_0_→^7^F_2_) from (Fig. 6-b), showed the broad UV band associated to the π -π* transition of PANI, revealing an energy transfer process between the polymeric matrix and Eu^3+^ ions; a small, sharp peak of Eu^3+^ at 394 nm (^7^F_0_→^5^L_6_) is observed, too. Hence, UV light excitation trigger the emissions of both base polymer and the Eu^3+^ ions. According to the conventional ‘antenna effect’ mechanism [21], luminescence under UV excitation typically involves the excitation of the singlet (S) state, followed by intersystem crossing (ISC) to the first triplet state (T) of the organic ligand, and subsequent energy transfer to the excited levels of the Ln^3+^ ion. Recent studies, however, identify direct energy transfer from the singlet state as a viable alternative that can significantly contribute to the overall quantum yield [22]. In this alternative pathway, energy is transferred to higher-lying excited levels—such as ^5^D_J_, ^5^L_6_, ^5^G_J_ [21]—by passing the triplet state, followed by non-radiative (NR) decay to the luminescent emitting levels and their characteristic emission (Fig. 6-c). We propose a similar process in the current system, wherein energy transfer from the high-energy singlet states of the ligands populates the Eu^3+^ ions excited levels, leading to characteristic emission (Fig. 6-c).

The estimation of the energy transfer efficiency from the analysis of the PL spectra recorded in undoped and Eu-doped PANI-EB results for about 60% indicating a relatively high sensitization of Eu³⁺ centers. However, the high ET efficiency is not accompanied by high luminescence signal because the Eu³⁺ ion is bound straight to the polymer chain and it undergoes rapid *non-radiative relaxation* from the excited state back into the semiconducting polymer chain (caused by high-energy vibrations of O-H and C = H bonds) reducing the overall photoluminescence quantum yield. In RE complexes the organic ligands surround and shield the ions blocking these quenching channels and boost internal quantum efficiencies [23].

Time-resolved luminescence spectroscopy measurements (Fig. 3S) revealed that Eu^3+^ luminescence lifetime (under direct 392 nm excitation) is longer than the value of 0.11 ± 0.02 ms reported for EuCl_3_⋅6H_2_O [24]. This increase is attributed to the suppression of non-radiative decay channels by the surrounding ligands. The intrinsic emission quantum efficiency can be computed from the PL spectra (i.e. ^5^*D*_0_ → ^7^*F*_0−4_ transitions) and the ^5^*D*_0_ → ^7^*F*_1_ transition as “*reference*” with an average index of 1.5 [25]. The emission quantum efficiency *η* of the emitting level is given by: *η* = *A*_*rad*_/*A*_*tot*_ where total decay rate *A*_*tot*_ = 1/*τ* and *τ* is the observed lifetime. The radiative decay rate *A*_*rad*_ =*A*_MD,0_ ·*n*^3^·(*I*_*tot*_*/I*_*MD*_) where *A*_MD,0_ is the spontaneous emission probability of the ^5^D_0_→^7^F_1_ transition (14.65 s^− 1^), *n* is the refractive index (1.5 for solid), and *I*_*tot*_/*I*_*MD*_ is the ratio of the total area of the Eu (III) luminescence spectrum to the area of the ^5^D_0_→^7^F_1_ transition [24] from which we obtain *A*_*rad*_ *=* 217 s^− 1^. As the occurrence of multiple emitting Eu^3+^ sites cannot be neglected the photoluminescence decay curve recorded at 614 nm (Fig. 3S) was analyzed by using two exponetial decays with amplitude (A) and *τ* characteristic time parameters: A_1_=560 and t_1_ = 0.25 ms with A_2_=542 and t_2_ = 0.13 ms. Acording to the analysis of kinetic experiments involving the relaxation of complex materials [26] we extracted an average time < *τ>*=0.21 ms. We obtained *A*_*tot*_ *=* 4762 (s^− 1^) from which emission quantum efficiency *η* ≅ 4%, a value much smaller than for other Eu^3+^-complexes ([5] and references therein). The relatively low luminescence intensity and quantum efficiency suggest the presence of other relaxation channels of the Eu^3+^ excited state as a competing process related to the high-energy vibrations of O-H and C = H bonds (within the polymeric chain).

Additional comments about the relatively can be made by estimation of the energy levels involved in the whole energy transfer processes. Assuming the optical band gap of PANI-EB at 325 nm (30.770 cm⁻¹) is associated with the singlet transition (S₁), the triplet state (T₁) can be estimated using a first-order approximation for π-π conjugated polymers [27]. Based on this approximation, the triplet state lies at approximately 430 nm (23200 cm^− 1^). According to the Latva’s empirical rule [28], for an efficient, sensitization (ligand-to-metal energy transfer), the optimal difference in energy between the triplet state T of the organic ligand and the emitting Eu^3+^ states should be within the range of 2.500 to 4.000 cm^− 1^. In the present case the energy difference corresponding to the energy transfer to ^5^D_0_ (at 17300 cm^− 1^) and ^5^D_1_ (at 21550 cm^− 1^) were about 5900 and 1650 cm^− 1^, respectively well outside the range limit. Hence, the energy transfer is not efficient because of the back-energy transfer from the ^5^D_1_ state to the ligand triplet state or the high energy gap for the ^5^D_0_ state, leading to a reduced photoluminescence quantum yield. In the present case, the energy gaps for transfer to the ^5^D_0_ (at 17300 cm^− 1^) and ^5^D_1_ (at 21550 cm^− 1^) levels are approximately 5,900 and 1,650 cm^− 1^, respectively—values that fall well outside the optimal range. Consequently, the energy transfer efficiency is hindered by either back-energy transfer from the ^5^D_1_ state to the ligand triplet state or the excessively large energy gap for the ^5^D_0_ state, ultimately resulting in a reduced energy transfer efficiency as we observed.

## Conclusion

We investigated the luminescence processes related to the complexation of the Eu^3+^ ions on the polyaniline emeraldine base (PANI-EB) particles surface, prepared by using liquid-phase impregnation method, through a detailed, multi-technique spectroscopic analysis. X-ray photoelectron spectroscopy (XPS) and Raman spectroscopy revealed Eu^3+^ ions interaction with the imine and amine groups of the PANI-EB. Photoluminescence measurements confirmed surface-mediated energy transfer within the complex via the *“antenna effect”*. However, the overall quantum yield remained relatively low due to limited energy transfer efficiency between the organic matrix and the lanthanide centers. 

## Supplementary Information

Below is the link to the electronic supplementary material.

Supplementary Material 1

## Data Availability

No datasets were generated or analysed during the current study.
