# Supplementary material for: Spectroscopic Investigations of the Surface-Level Interaction of Europium (III) Ions and Polyaniline Particles
Source: J Fluoresc. 2026 Jul 30;36(8):5113–20. doi: 10.1007/s10895-026-04897-z (PMC13553658; doi:10.1007/s10895-026-04897-z)
Supplement: Supplementary file 1 [file 10895_2026_4897_MOESM1_ESM.docx]

**Supplementary information**

**Fig. 1S.** The EDX elemental analysis of Eu^3+^-doped PANI-EB particles.

**Fig. 2S.** EPR spectra recorded in the X-band (9.86GHz) on Eu^3+^-doped PANI-EB particles.

**Fig. 3S.** The Eu^3+^ luminescence decay curve recorded at 614 nm represented in logarithmic scale; the two exponentail decays are represented by dotted lines
